# Beneficial Effects of *Asparagus officinalis* Extract Supplementation on Muscle Mass and Strength following Resistance Training and Detraining in Healthy Males

**DOI:** 10.3390/sports11090175

**Published:** 2023-09-05

**Authors:** Barakat Denben, Siriporn Sripinyowanich, Ratree Ruangthai, Jatuporn Phoemsapthawee

**Affiliations:** 1 Department of Sports Science and Health, Faculty of Sports Science, Kasetsart University, Nakhon Pathom 73140, Thailand; barakat.d@ku.th (B.D.); ratree.r@ku.th (R.R.); 2 Department of Science and Bioinnovation, Faculty of Liberal Arts and Science, Kasetsart University, Nakhon Pathom 73140, Thailand; siriporn.srip@ku.th

**Keywords:** 20-hydroxyecdysone, muscle strength, resistance training, detraining, insulin-like growth factor-1

## Abstract

The phytoecdysteroid 20-hydroxyecdysone (20E) is widely used for resistance training (RT). Little is known about its potential ergogenic value and detraining effects post-RT. This study aimed to examine the effects of 20E extracted from *Asparagus officinalis* (*A. officinalis*) on muscle strength and mass, as well as anabolic and catabolic hormones following RT and detraining. Twenty males, aged 20.1 ± 1.1 years, were matched and randomly assigned to consume double-blind supplements containing either a placebo (PLA) or 30 mg/day of 20E for 12 weeks of RT and detraining. Before and after RT and detraining, muscle strength and mass and anabolic and catabolic hormones were measured. This study found that 20E reduced cortisol levels significantly (*p* < 0.05) compared to the PLA, yet no effect was observed on muscle mass, strength, or anabolic hormones after RT. Subsequent to 6 weeks of detraining, the 20E demonstrated a lower percentage change in 1RM bench press/FFM than the PLA (*p* < 0.05). Compared to the PLA, detraining throughout the 12 weeks resulted in a lower percentage change in thigh (*p* < 0.05) and chest (*p* < 0.01) circumferences, as well as reduced cortisol levels (*p* < 0.01), with 20E. Our findings demonstrate that 20E supplementation is a promising way to maintain muscle mass and strength during detraining. Accordingly, 20E may prevent muscle mass and strength loss due to detraining by lowering catabolic hormone levels.

## 1. Introduction

Low muscle strength is associated with lower functional capacity, and it has been shown that muscle strength is inversely related to the risk of mortality and cardiovascular disease [1]. Loss of muscle mass also causes a decrease in metabolic rate and, as a result, increases body fat, which is linked to a variety of diseases and adverse metabolic conditions [2]. Maintaining muscle strength and mass is essential for maintaining independence and lowering the risk of mortality and illness. 

Resistance training (RT) is a well-established method of exercise for increasing muscular strength and size. Regular RT increases muscular strength and hypertrophy via mechanical [3], metabolic [4], and hormonal [5] stimuli, which regulate gene expression and protein synthesis [6]. On the other hand, training interruptions, caused by a variety of planned and unplanned factors ranging from illness to vacations, can lead to a loss of muscle strength and mass [7]. Nevertheless, using dietary supplements may lessen the effects of detraining. There has been a great deal of emphasis in recent years on the development of dietary supplements that improve physical performance, particularly muscular strength and mass. 

One interesting type of supplement is ecdysteroids, also known as phytoecdysteroids. They are abundant in a wide range of plant species, such as *Leuzea carthamoides alias*, *Rhaponticum carthamoides*, *Rhaponticum integrifolium*, etc. [8,9], as well as in the widely consumed spinach (*Spinacia oleracea*) [10]. The phytoecdysteroid 20-hydroxyecdysone (20E), also known as ecdysterone, is the most common phytoecdysteroid used as an ergogenic aid, and it is often the most abundant phytoecdysteroid in the plant [11]. Notably, 20E affects major metabolic pathways, including protein synthesis [10]. The hard-stem by-product of asparagus (*A. officinalis*) contains significant amounts of 20E, according to phytochemical analyses of traditional medicinal plants from around the world [11,12]. Asparagus is a low-calorie herbaceous perennial vegetable that is both nutritionally and commercially important. The asparagus stem and root, which account for 76.5% of its weight, are discarded as waste [13]. The hard-stem by-product contained 20E at relatively high levels of approximately 2.34 mg/g dry weight [12]. As a result, promoting the hard-stem by-product as a dietary supplement would increase the value of the wasted asparagus. 

One of the most intriguing properties of 20E in mammals is its anabolic effect, which is similar to that of anabolic steroids, yet it lacks an androgenic effect [14] and has low toxicity [15,16]. Extensive research on the potential anabolic-promoting effects of ecdysterone in various animal models and cell cultures has been reported [10,14,17,18,19,20]. In contrast to cell culture and animal research, supplementation with ecdysterone to enhance performance has not yet been widely studied in humans. Only two studies have examined the effects of ecdysterone supplementation on training adaptations and anabolic and catabolic markers in resistance-trained men [21] and young men [15]. A previous human study suggested that supplementing with 200 mg/day of 20E for 8 weeks had no effect on lean mass, muscle strength, or muscle power adaptations, nor did it affect the anabolic or catabolic hormone status of resistance-trained men [21]. However, a recent study involving young men found that supplementing with 48 mg/day of ecdysterone for 10 weeks increased muscle mass and strength [15]. Furthermore, its detraining effects following RT are unknown. 

Understanding the effects of 20E supplementation on muscle mass and strength after training and subsequent detraining periods will help us to optimize muscle strength and delay mass loss. The purpose of this study was to examine the effects of 20E extracted from *A. officinalis* on muscle strength and mass after RT and detraining. A second objective of this study was to examine the effects of 20E extracted from *A. officinalis* on anabolic and catabolic hormones following RT and detraining, because the balance of these hormones is essential for maintaining muscle strength and mass.

## 2. Materials and Methods

### 2.1. Participants and Study Design

Twenty healthy males volunteered to participate in the present study (age = 20.1 ± 1.1 years; body mass = 72.2 ± 9.7 kg; height = 171.9 ± 4.8 cm; fat-free mass = 55 ± 6.3 kg; peak oxygen consumption = 40.6 ± 7.7 mL/kg/min). Participants’ characteristics are presented in Table 1. Prior to the start of the study, each participant filled out a medical history and activity questionnaire. All participants lacked a minimum of one year of RT experience and had not engaged in RT in the six months prior to the study’s outset. The participants were free of major metabolic disorders (e.g., heart disease, diabetes, thyroid disease, etc.) and major musculoskeletal disorders that would limit their ability to exercise and/or complete the tests. In addition, none of the participants were taking any medications, nutritional supplements, or ergogenic dietary supplements (i.e., creatine, androstenedione, myostatin inhibitors, prohormones, etc.) that could have confounded the study’s findings. The study was approved by the Kasetsart University Research Ethics Committee (COA. No. COA64/050). The participants were informed of the benefits and risks of the investigation and required to sign an approved consent form outlining the risks of the experiment prior to participation. 

**Figure 1:**
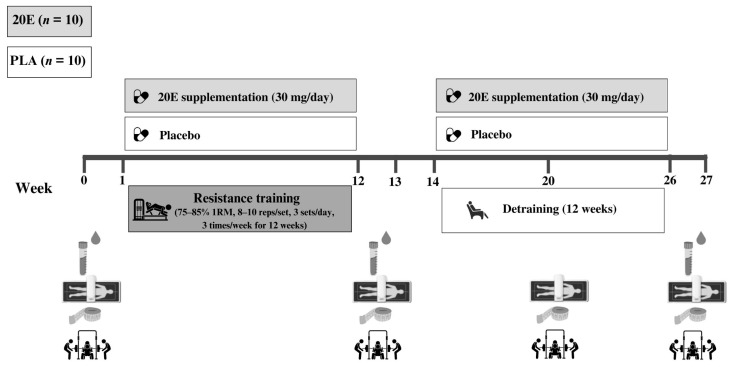
Experimental design. The training periods were split into three blocks of four weeks, each with a specific number of sets and repetitions. The training intensity load of each block is indicated in % of 1RM. Following training and detraining, blood collection and 1RM tests were performed ~72 h after the last RT session. PLA, placebo group; 20E, 20-hydroxyecdysone supplementation group; 1RM, one-repetition maximum. White box: PLA group (*n* = 10); grey box: 20E group (*n* = 10).

This study was a randomized, double-blind, placebo-controlled trial, as depicted schematically in Figure 1. The participants were randomly assigned to one of two groups: supplementation with 20E extracted from the hard-stem by-product of *A. officinalis* (20E; *n* = 10) or placebo (PLA; *n* = 10). A 20E capsule of 500 mg of *A. officinalis* extract contained 32.2 mg/g dry weight of 20E. The 20E group consumed approximately 30 mg of 20E per day, equivalent to 0.41 mg/kg BW in a volunteer of 73 kg. Conversely, the PLA group consumed 2 capsules of maltodextrin per day, which were virtually identical in size, shape, and color. All participants took the two capsules with their morning meals and received 12 weeks of supplementation with an RT period, followed by a 12-week detraining period. All participants were instructed to maintain their normal levels of daily activity throughout the 24 weeks of the study. They were also instructed to maintain their regular diet throughout the duration of the study, and they completed a 3-day dietary recall to determine their nutritional status, which was accounted for in the analysis of dietary intake. All dependent variables were assessed prior to training, after 12 weeks of training, and after 6 and 12 weeks of detraining.

### 2.2. Measurements

#### 2.2.1. Anthropometrics and Body Composition

Body weight was measured via an electronic scale (Filizzola PL 150, Filizzola^®^ Ltd., São Paulo, Brazil). Height was measured minus shoes using a standard stadiometer (Health o Meter™ Professional, Sunbeam Products Inc., Boca Raton, FL, USA). Body mass index (BMI) was calculated as weight divided by height squared. Body composition and fat distribution were estimated using dual-energy X-ray absorptiometry (Lunar iDXA, GE Healthcare, Chicago, IL, USA). Participants were then positioned and scanned according to the manufacturer’s standard specifications in a climate-controlled room. Total body lean mass, fat mass, and regional fat mass of the total body, trunk, arms, and legs were analyzed utilizing the enCore software (V17 software, Lunar iDXA, GE Healthcare, Chicago, IL, USA).

#### 2.2.2. Body Circumferences

Body girth was measured twice at six locations (chest, waist, arm, forearm, thigh, and calf) on the right side of the body with a flexible inelastic tape. To give the skin enough time to return to its normal texture, the measurement sites were rotated for the second measurement. If the duplicate measurements were not within 5 mm, another measurement was taken. Our findings showed excellent internal consistency (alpha coefficient = 1.00).

#### 2.2.3. Blood Samples and Analysis

All measurements were performed following 12 h of fasting at baseline and 72 h after the last training session to minimize any acute effects of exercise. All blood specimens without anticoagulant were centrifuged at 4244× *g* for 10 min, with the resultant serum specimens aliquoted and stored at −20 °C. The serum levels of insulin-like growth factor-1 (IGF-1), free testosterone, and cortisol were measured using commercial assays (Shenzhen New Industries Biomedical Engineering Co., Ltd., Shenzhen, China) on a chemiluminescence immunoassay analyzer from the same manufacturer. Sandwich CLIA was employed to measure IGF-1, while competitive CLIA was used to measure free testosterone and cortisol. The measuring ranges specified by the manufacturer for IGF-1, testosterone, and cortisol were 2.5–2000 ng/mL, 0.5–150 pg/mL, and 2.5–600 ng/mL, respectively. For results below this range, the measuring range’s lowest point was applied instead for calculations. A precision verification study was conducted according to the CLSI EP5-A2 protocol. The maximum coefficient of variation for assays of IGF-1, free testosterone, and cortisol was found within the limits claimed by the manufacturer. The activities of pathophysiological enzymes such as serum aspartate transaminase (AST), alanine transaminase (ALT), alkaline phosphatase (ALP), blood urea nitrogen (BUN), and creatinine were assayed using commercial kits employing a clinical chemistry analyzer (BS-360E, Mindray Bio-Medical Electronics Co., Ltd., Shenzhen, China).

#### 2.2.4. Muscular Strength

The one-repetition maximum (1RM) was determined using an indirect method on the leg press, followed by bench presses, as per National Strength and Conditioning Association (NSCA) guidelines [22]. Before the 1RM test, the participants performed a warm-up consisting of one set of ten repetitions with a light load, which they could perform 12 to 15 times. In the 1RM testing, if any participant could perform more than 10 repetitions in an attempt, the load was increased by 30 pounds for the leg press and 10 pounds for the bench press. The rest period between each attempt was 3 min. The workload of 1RM was calculated for each participant based on the loads and repetitions that participants were able to perform using the 1RM table. Although cadence was uncontrolled, the participants were asked to control the eccentric and concentric movements. All assessments were supervised by an NSCA-certified strength and conditioning specialist.

### 2.3. Training and Detraining Protocols

Participants performed a 5-min walk on a treadmill before RT and a 5-min whole-body stretch before and after training. The RT program consisted of nine exercises (back squat, chest press, leg extension, seated row, leg curl, shoulder press, calf raises, lat pulldown, and plank) performed three times per week for 12 weeks with at least 48 h of rest between training sessions. The program consisted of 3 sets of 8–10 repetitions with as much weight as participants could perform per set (typically 75–80% of 1RM), 60-s rest periods between sets, and 60-s rest periods between exercises. Each repetition consisted of a controlled concentric contraction and a 2-s eccentric contraction. The participants increased their training weight by 1–2.5 kg for upper-body muscles and 2.5–5 kg for lower-body muscles each week under supervision, beginning with an intensity of 75% of their 1RM. If this could not be guaranteed, the weight was decreased or not increased in accordance with the plan. The consistency index for the RT program was 0.87–1. All training took place in the fitness center of the Faculty of Sports Science at Kasetsart University. All routines were directly supervised by personal trainers to ensure their correctness. The participants recorded the amount of weight lifted and the number of repetitions performed for each set on their training cards, allowing for the determination of the training volume. The research assistants also signed the participant training cards to confirm the participants’ attendance and workout completion.

The participants were instructed to resume their normal lifestyles after completing the RT program and to avoid any form of regular exercise for a 12-week period. They were contacted on a regular basis during this time to ensure that they did not engage in any other form of regular exercise or make any other lifestyle changes during the 12 weeks of detraining.

### 2.4. Preparation of Plant Materials and Determination of 20E Content

The hard-stem by-products of *A. officinalis* were collected from the farmer’s fields in Nakhon Pathom, Thailand, and sent to the laboratory within 5 h until processing. The fresh asparagus by-product was washed in clean running water and rewashed for 10 min in an ultrasonic bath. The plant material was then chopped into 5-mm-long spear pieces and oven dried for 30 h at 60 °C to achieve a consistent weight (moisture content 5% *w*/*w*). The dry tissue was homogenized into a powder with a mechanical grinder and screened through an 850-mm sieve aperture. The powdered asparagus by-product (300 g) was extracted with 95% EtOH (1.5 L) for 3 days, repeated twice. The extract was filtered and evaporated to dryness. The obtained residue was resuspended in water and then analyzed using HPLC chromatography on a C18 Sep-Pak cartridge (2.1 mm × 50.0 mm, 1.8 mm, Agilent Technologies, Waldbronn, Germany) at 40 °C with a flow rate of 1 mL/min and a mobile phase:acetonitrile/H_2_O ratio of 20:80 in 20 min. The 20E was determined by monitoring UV absorbance at 245 nm and characterized by its UV spectrum and retention time. The identification of each compound was based on a combination of retention time and spectral matching. The chromatograms were adjusted graphically to simulate a match for the 20E retention time at 25.923 min, as depicted in Figure S1. The quantification of 20E was carried out using a previously described method [23,24]. In brief, standard curves were generated using HPLC-grade >93% purified 20E (Sigma-Aldrich, St. Louis, MO, USA). Calibration of the system with known quantities of these molecules enabled the determination of the concentration of 20E in the asparagus by-product based on standard samples (Sigma-Aldrich, St. Louis, MO, USA). The stock solutions of the standards were prepared and applied in triplicate to the HPLC. The peak areas were recorded, and calibration curves were prepared by plotting the peak areas against concentrations.

For capsule preparation, the dried ground asparagus by-product was filled into capsules under aseptic conditions and controlled by their weight of 500 mg per capsule. Then, twenty capsules were picked at random and weighted individually with an analytical balance to control the weight. Each capsule contained 32.2 mg/g dry weight 20E. The capsules were then stored at −20 °C for further experiments.

### 2.5. Statistical Analyses

The results were presented as the mean ± standard deviation (SD). The Shapiro–Wilk test was conducted to examine the normality of the data. An unpaired t-test was employed to determine the differences at baseline between groups. A two-way analysis of variance with repeated measures [group (the PLA and the 20E groups) × time (baseline, following 12 weeks of training, and 6 weeks and 12 weeks of detraining)] was used to determine the effects of 20E supplementation and time on dependent variables. If a significant interaction or main effect was noted, univariate analysis was applied for post-hoc comparisons. An effect size (ES) analysis was performed using eta-squared (*η*^2^) for the two-way ANOVA, which interpreted 0.01, 0.06, and 0.14 as small, medium, and large, respectively. Statistical significance was set at *p* < 0.05. Statistical analyses were conducted using SPSS version 26 for Windows (SPSS Inc., Chicago, IL, USA). 

## 3. Results

Participants’ characteristics did not differ significantly between groups. Across all baseline parameters examined, all groups exhibited similar profiles (see Table 1). During experimentation, the average energy intake did not differ significantly between groups (Table S1). In the 20E group, protein accounted for approximately 19% of daily energy intake, while carbohydrates and fat accounted for 50.4% and 30.6%, respectively. In the PLA group, protein accounted for approximately 19.4% of daily energy intake, while carbohydrates and fat accounted for 50.4% and 30.2%, respectively. Throughout the duration of the experiment, these percentages did not significantly alter for either group. Aside from the interventions, there was no significant change in habitual physical activity over time, and no difference was found between groups. The exercise attendance rates in the PLA and the 20E groups were 99.3% and 100%, respectively. During the training period, both the PLA and the 20E participants completed 100% of the prescribed exercise volume. The hard-stem products of *A. officinalis* extract showed neither adverse effects nor significant changes in liver or kidney enzymes throughout the study.

### 3.1. Body Composition

The two-way ANOVA revealed no statistically significant interaction between groups and times for BM, %BF, FM, FFM, or LM. Nonetheless, the main effects analysis revealed that time had a statistically significant effect on total BM (F_(3,54)_ = 3.216, *p* = 0.030, *η*^2^ = 0.152), total FFM (F_(3,54)_ = 6.637, *p* < 0.001, *η*^2^ = 0.278), arm mass (F_(3,54)_ = 5.020, *p* = 0.004, *η*^2^ = 0.218), arm fat percentage (F_(3,54)_ = 3.854, *p* = 0.014, *η*^2^ = 0.176), arm LM (F_(3,54)_ = 8.077, *p* < 0.001, *η*^2^ = 0.310), and leg LM (F_(3,54)_ = 3.346, *p* = 0.026, *η*^2^ = 0.157). Pairwise post-hoc tests revealed that 12 weeks of training resulted in significantly increased total FFM (*p* < 0.05) and arm LM (*p* < 0.05) and a significantly decreased arm fat percentage (*p* < 0.05) compared to pre-training in the 20E (Table 2). After the detraining period, no significant changes in body composition were observed following detraining in the 20E. Meanwhile, total BM (*p* < 0.05), arm mass (*p* < 0.05), and leg LM (*p* < 0.05) decreased significantly following 12 weeks of detraining compared to 12 weeks of training in the PLA (Table 2). 

**Figure 2:**
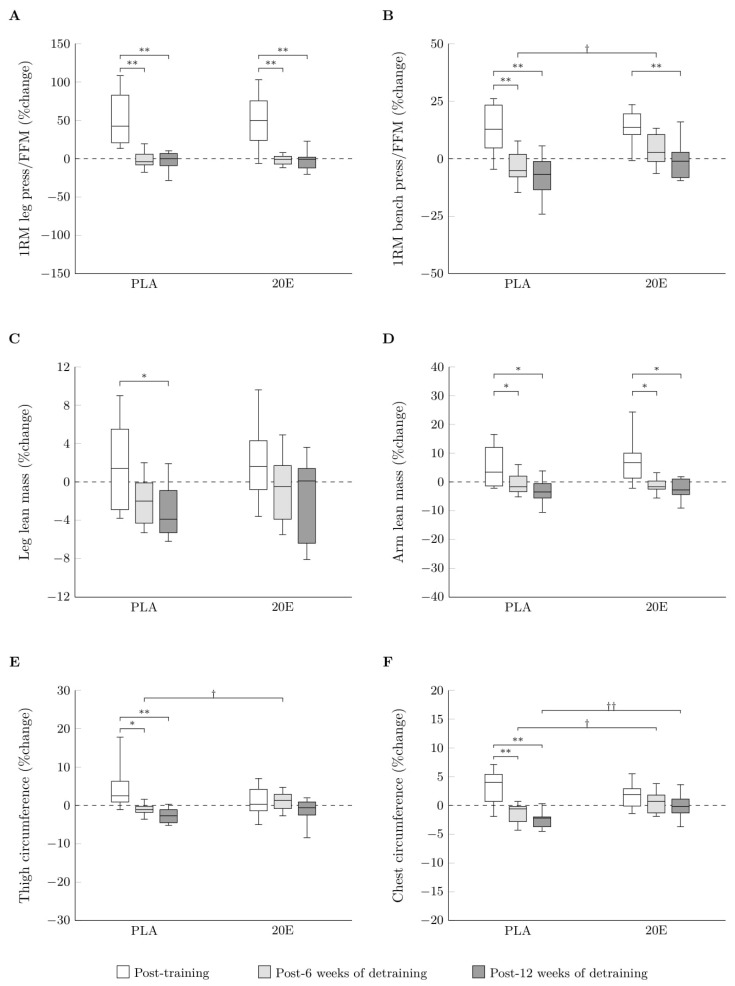
Boxplots for the percentage changes from baseline and after 12 weeks of training of 1RM of leg press/FFM (**A**), 1RM of bench press/FFM (**B**), leg lean mass (**C**), arm lean mass (**D**), thigh circumference (**E**), and chest circumference (**F**) in the PLA (*n* = 10) and 20E groups (*n* = 10). The upper, middle, and bottom horizontal lines of each box represent the 1st quartile, median, and 3rd quartile of the data, respectively, and the error bars indicate the minimum and maximum values. PLA, placebo group; 20E, 20-hydroxyecdysone supplementation group; 1RM, one-repetition maximum; FFM, fat-free mass. * *p* < 0.05 and ** *p* < 0.01 to within-group comparison. † *p* < 0.05 and †† *p* < 0.01 to between-group comparison.

Figure 2 shows boxplots for the percentage changes from baseline and after 12 weeks of training of leg LM and arm LM in the PLA and 20E groups. The two-way ANOVA revealed that there was no statistically significant interaction between groups and times for percentage changes in leg LM or arm LM. Notwithstanding, the main effects analysis revealed that time had a statistically significant effect on percentage changes in leg LM (F_(2,36)_ = 7.502, *p* = 0.002, *η*^2^ = 0.294) and arm LM (F_(2,36)_ = 16.708, *p* < 0.001, *η*^2^ = 0.481). Pairwise post-hoc tests revealed that following 12 weeks of detraining, the leg LM was significantly lower (*p* < 0.05) than after 12 weeks of training in the PLA, whereas no significant change in leg LM was observed in the 20E following the detraining period (Figure 2C). In addition, after 6 and 12 weeks of detraining, there was a significant decrease in arm LM percentage compared to after 12 weeks of training in both the PLA (*p* < 0.05 and *p* < 0.05, respectively) and the 20E (*p* < 0.05 and *p* < 0.05, respectively)—see Figure 2D.

### 3.2. Body Circumferences

The two-way ANOVA with a medium *η*^2^ ES revealed a trend towards interactions between groups and times for chest circumference (F_(3,54)_ = 2.757, *p* = 0.051, *η*^2^ = 0.133). There was, however, no significant chest circumference variance between groups. Furthermore, for waist, arm, forearm, thigh, and calf circumferences, no statistically significant interaction between groups and times was found. The main effects analysis revealed that time had a statistically significant effect on chest (F_(3,54)_ = 10.211, *p* < 0.001, *η*^2^ = 0.362), arm (F_(3,54)_ = 3.876, *p* = 0.014, *η*^2^ = 0.177), forearm (F_(3,54)_ = 5.563, *p* = 0.002, *η*^2^ = 0.236), and thigh (F_(3,54)_ = 5.594, *p* = 0.002, *η*^2^ = 0.237) circumferences. Pairwise post-hoc tests also revealed that 12 weeks of training resulted in significantly increased chest circumference compared to pre-training in the PLA (*p* < 0.01) and the 20E (*p* < 0.05) (Table 2). After the detraining period, chest (*p* < 0.01), forearm (*p* < 0.01), and thigh (*p* < 0.05) circumferences decreased significantly after 12 weeks of detraining compared to 12 weeks of training only in the PLA, whereas no significant changes in body circumferences were observed following detraining in the 20E (Table 2). 

Figure 2 shows boxplots for the percentage changes from baseline and after 12 weeks of training for thigh and chest circumferences in the PLA and 20E groups. The two-way ANOVA revealed a statistically significant interaction between group and time for the percentage changes in thigh (F_(2,36)_ = 3.686, *p* = 0.035, *η*^2^ = 0.170) and chest (F_(2,36)_ = 4.865, *p* = 0.013, *η*^2^ = 0.213) circumferences. After 12 weeks of detraining, the 20E demonstrated a lower percentage alteration in thigh circumference (*p* < 0.05) than the PLA (Figure 2E). Chest circumference, as a percent change from after 12 weeks of training, was significantly lower for the 20E compared to the PLA at 6 (*p* < 0.05) and 12 (*p* < 0.01) weeks of detraining (Figure 2F). Furthermore, the main effects analysis revealed that time had a statistically significant effect on the percentage changes in thigh (F_(2,36)_ = 8.187, *p* = 0.001, *η*^2^ = 0.313) and chest (F_(2,36)_ = 15.966, *p* < 0.001, *η*^2^ = 0.470) circumferences. Pairwise post-hoc tests revealed that subsequent to 12 weeks of detraining, the percentage changes in thigh (*p* < 0.01) and chest (*p* < 0.01) circumferences were significantly lower than after 12 weeks of training in the PLA. Notwithstanding, there was no statistically significant difference in the percentage changes in thigh or chest circumferences in the 20E following detraining compared to after 12 weeks of training (Figure 2E,F).

### 3.3. Lower- and Upper-Body Muscle Strengths

The two-way ANOVA revealed that for lower-body or upper-body muscle strength, there was no statistically significant interaction between group and time. However, the main effects analysis revealed that time had a statistically significant effect on 1RM leg press (F_(3,54)_ = 32.145, *p* < 0.001, *η*^2^ = 0.593), 1RM leg press/FFM (F_(3,54)_ = 36.968, *p* < 0.001, *η*^2^ = 0.673), 1RM bench press (F_(3,54)_ = 29.654, *p* < 0.001, *η*^2^ = 0.622), and 1RM bench press/FFM (F_(3,54)_ = 17.100, *p* < 0.001, *η*^2^ = 0.487). Pairwise post-hoc tests revealed that 12 weeks of training resulted in a significantly increased 1RM leg press (*p* < 0.01 and *p* < 0.01, respectively), 1RM leg press/FFM (*p* < 0.01 and *p* < 0.01, respectively), 1RM bench press (*p* < 0.01and *p* < 0.01, respectively), and 1RM bench press/FFM (*p* < 0.01 and *p* < 0.01, respectively) compared to pre-training in the PLA and the 20E, respectively (Table 2). After the detraining period, there were no significant changes in any lower-body or upper-body muscle strengths in the PLA or the 20E after detraining, except in the 1RM bench press (*p* < 0.01), which was significantly decreased after 12 weeks of detraining only in the PLA (Table 2).

Figure 2 shows boxplots for the percentage changes from baseline and after 12 weeks of training in lower-body or upper-body muscle strength in the PLA and 20E groups. The two-way ANOVA revealed a statistically significant interaction between group and time for the 1RM bench press/FFM percentage change (F_(2,36)_ = 9.240, *p* = 0.005, *η*^2^ = 0.406). Following 6 weeks of detraining, the 20E exhibited a lower percentage change in 1RM bench press/FFM (*p* < 0.05) than the PLA (Figure 2B). Furthermore, the main effects analysis revealed that time had a statistically significant effect on the percentage changes in 1RM leg press/FFM (F_(2,36)_ = 8.187, *p* = 0.001, *η*^2^ = 0.313) and 1RM bench press/FFM (F_(2,36)_ = 22.101, *p* < 0.001, *η*^2^ = 0.551). Pairwise post-hoc tests revealed that after 6 and 12 weeks of detraining, the percentage changes in 1RM leg press/FFM (*p* < 0.01 and *p* < 0.01, respectively) and 1RM bench press/FFM (*p* < 0.01 and *p* < 0.01, respectively) were significantly lower than after 12 weeks of training in the PLA. Subsequent to 12 weeks of detraining, the percentage changes in 1RM leg press/FFM and 1RM bench press/FFM (*p* < 0.01 and *p* < 0.01, respectively) in the 20E group were significantly lower than after 12 weeks of training. However, there was no statistically significant difference in the percentage changes in 1RM bench press/FFM in the 20E after 6 weeks of detraining compared to after 12 weeks of training (Figure 2A,B).

### 3.4. Anabolic and Catabolic Hormones

**Figure 3:**
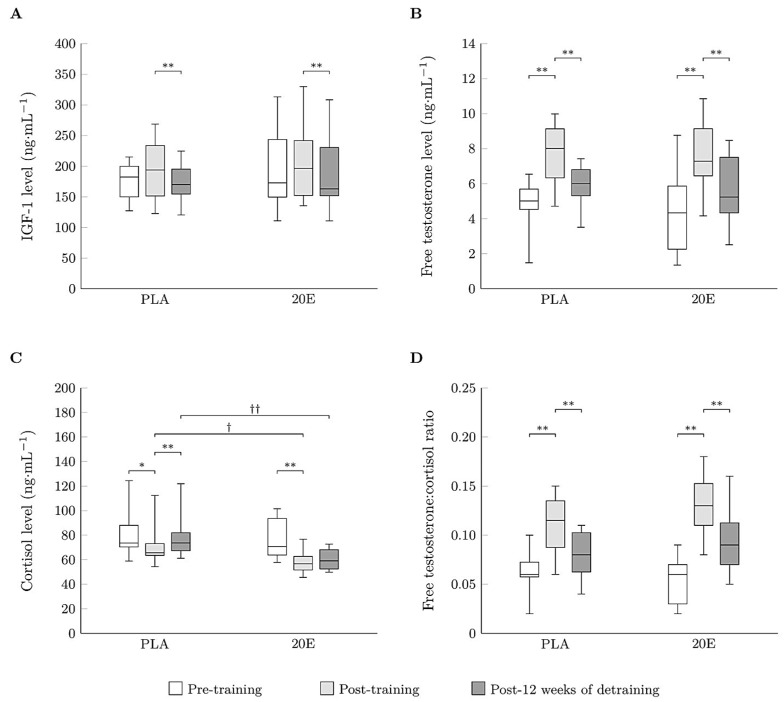
Boxplots for the levels of IGF-1 (**A**), free testosterone (**B**), cortisol (**C**), and free testosterone:cortisol ratio (**D**) before training, after 12 weeks of training, and after detraining in the PLA (*n* = 10) and 20E groups (*n* = 10). The upper, middle, and bottom horizontal lines of each box represent the 1st quartile, median, and 3rd quartile of the data, respectively, and the error bars indicate the minimum and maximum values. PLA, placebo group; 20E, 20-hydroxyecdysone supplementation group; IGF-1, insulin-like growth factor 1. * *p* < 0.05 and ** *p* < 0.01 to within-group comparison. † *p* < 0.05 and †† *p* < 0.01 to between-group comparison.

The two-way ANOVA revealed a statistically significant interaction between group and time for the cortisol level (F_(2,36)_ = 8.656, *p* = 0.001, *η*^2^ = 0.325) and free testosterone to cortisol (fTC) ratio (F_(2,36)_ = 6.131, *p* = 0.005, *η*^2^ = 0.254). The 20E presented lower levels of cortisol than the PLA after 12 weeks of training (*p* < 0.05) and detraining (*p* < 0.01); see Figure 3C. There was no statistically significant variance between groups in terms of IGF-1, free testosterone, or the fTC ratio (Figure 3). The main effects analysis revealed that time had a statistically significant effect on levels of IGF-1 (F_(2,36)_ = 7.500, *p* = 0.002, *η*^2^ = 0.294), free testosterone (F_(2,36)_ = 68.333, *p* < 0.001, *η*^2^ = 0.792), and cortisol (F_(2,36)_ = 33.080, *p* < 0.001, *η*^2^ = 0.648) and the fTC ratio (F_(2,36)_ = 106.325, *p* < 0.001, *η*^2^ = 0.855). Pairwise post-hoc tests revealed that 12 weeks of training resulted in significantly greater levels of free testosterone (*p* < 0.01 and *p* < 0.01, respectively) and the fTC ratio (*p* < 0.01 and *p* < 0.01, respectively) and lower levels of cortisol (*p* < 0.05 and P < 0.01, respectively) in both the PLA and the 20E groups compared to pre-training (Figure 3). In both the PLA and the 20E groups, levels of IGF-1 (*p* < 0.01 and *p* < 0.01, respectively) and free testosterone (*p* < 0.01 and *p* < 0.01, respectively) and the fTC ratio (*p* < 0.01 and *p* < 0.01, respectively) were significantly lower after 12 weeks of detraining compared to 12 weeks of training. Cortisol levels were significantly higher (*p* < 0.01) following 12 weeks of detraining compared to 12 weeks of training in only the PLA. However, no significant changes in cortisol levels were observed in the 20E post-detraining (Figure 3).

## 4. Discussion

In recent years, 20E has gained attention due to its potential health benefits and its presence in a variety of edible plants, such as spinach (*Spinacia oleracea* L.) [9], ginseng (*Panax ginseng* C.A. Mey) [9], quinoa (*Chenopodium quinoa* Willd.) [10,25], and also asparagus (*A. officinalis*) [25,26,27]. Moreover, 20E has been detected in the leaves, stems, and roots of asparagus [26] and even achieved in the hard-stem ends of asparagus, which account for around 30–40% of each spear and are typically discarded as by-products. The first is removed during industrial processing, while the second is an agricultural by-product. This by-product has been reported to be rich in many bioactive phytochemicals [9], especially 20E [11]. In the current study, we therefore prepared a supplement from the hard-stem by-product of asparagus.

The objective of this study was to determine the effects of supplementation with 30 mg/day of 20E extracted from the hard-stem by-products of *A. officinalis* on muscle strength and mass, as well as anabolic and catabolic hormone responses, in healthy men following training and detraining. The findings of this study indicate that the administration of 20E supplementation yields positive outcomes in terms of anticatabolic hormone levels. However, it does not appear to have any impact on body circumference, muscle strength, or the responses of anabolic hormones during the RT period. Although 20E had a positive time effect on total FFM, arm LM, and arm fat percentage after the RT period, there were no significant differences between the PLA and 20E groups when these parameters were compared. Nonetheless, 20E supplementation rendered positive effects on body circumferences (% changes in thigh and chest circumferences), upper-body muscle strength (% changes in 1RM bench press/FFM), and anticatabolic hormonal effects during the detraining period. Furthermore, no significant changes in body composition or muscle mass during the detraining period were discovered in the 20E group. These results support the claim that supplementation with 20E increases muscle mass during RT and may also delay the loss of muscle mass and strength during detraining. In addition, no direct side effects of supplementation with 20E were reported. No negative effect on BUN, creatinine, AST, ALT, or ALP was discovered either. 

During the RT period, supplementation with 30 mg/day of 20E increased total FFM by 2.8%, arm LM by 5.2%, and arm fat percentage by 1.5% in healthy men. In these conditions, the 20E group demonstrated a positive time effect. Similar positive effects on body composition and muscle hypertrophy have been reported with both low (12 mg/day) and high (48 mg/day) doses of ecdysterone supplementation over 10 weeks of RT in young men [15]. Furthermore, these findings are consistent with previous research on the effects of anabolic steroid (testosterone and estradiol) supplements [28,29,30]. Although the anabolic effects of 20E are similar to the hypertrophic effects of testosterone, the effects are not as strong as those of testosterone supplementation [31].

Several studies [14,15,19] have demonstrated that 20E supplementation has anabolic effects in vitro by increasing protein synthesis in skeletal muscle cells (C2C12 cells). In addition, the positive effect of 20E supplementation has been demonstrated in numerous animal experiments [18,20,32]. Moreover, 20E supplementation can increase anabolic activity in skeletal muscle as well as cell proliferation and growth, thus bringing about an increase in muscle mass. As a result, similar to cell culture and animal studies, 20E supplementation has a positive anabolic effect on muscle growth in humans. It is possible that the faster rate of muscle mass gain, especially in the upper-body muscles, with 20E supplementation over a longer duration of resistance training would result in greater increases in muscle strength.

However, when comparing muscle mass development after the RT period, this study found no significant differences between the PLA and the 20E groups. These results contrast with those of Isenmann et al. [15], who discovered differences between the groups receiving high doses (48 mg/day) of ecdysterone and a placebo group. The contrasting observations can be attributed to the varying concentrations of ecdysterone and leucine (100 mg per capsule). Isenmann et al. [15] used a dietary supplement containing 100 mg of ecdysterone derived from spinach extract and 100 mg of leucine. Leucine administration has been shown to improve skeletal muscle performance. Both factors are important and can result in varying observations following the RT period. 

To our knowledge, no previous studies have investigated the effects of 20E supplementation on muscle mass after detraining. In the PLA group, percentage changes in arm LM, thigh circumference, and chest circumference decreased by 0.7%, 1.2%, and 1%, respectively, whereas, in the 20E group, percentage changes were found only in arm LM, which decreased by 1.3% during the 6-week detraining. Moreover, leg LM, arm LM, thigh circumference, and chest circumference decreased during the 12-week detraining period by 3.2%, 3.2%, 2.7%, and 2.6%, respectively, in the PLA, whereas, in the 20E, only arm LM decreased by 2.3%.

An interesting result of this study was that dietary supplementation with 20E rendered positive effects on percentage changes in thigh and chest circumferences and maintained changes in muscle mass throughout the 12 weeks of detraining. Although the mechanism of this anabolic effect after detraining is not fully understood, 20E appears to decrease protein breakdown [18] and increase protein synthesis [14]. In addition, a previous study demonstrated that 20E attenuated tenotomy-induced muscle atrophy in predominantly slow-twitch muscle in rat skeletal muscle [18]. Consequently, 20E could be useful as a supplement to prevent muscle loss caused by detraining. 

Although the precise mechanism underlying the anabolic effects is unknown, it has been proposed that estrogen receptor beta activation mediates ecdysterone’s anabolic activity [19,33]. Antiestrogen, but not antiandrogen, could counteract ecdysterone’s hypertrophic effect, which could be due to the modulation of phosphorylation effects [19,33]. Furthermore, the phosphorylation cascade of phosphatidylinositol 3-kinase (PI3K)/protein kinase B (Akt) has been shown to be involved in ecdysterone’s hypertrophic activity in C2C12 cells [34]. 

In terms of muscular strength, both the PLA and the 20E groups improved their lower- and upper-body muscular strength significantly. A linear periodization model was applied for the training intervention, which systematically increases the weight to maximize strength. The improvement in lower- and upper-body muscle strength in each group is similar to what was seen in previous studies [15,35] that used a linear periodization model with and without ecdysterone supplementation. This means that the conceptual training design produces comparable results to the previous studies. This result is consistent with previous research on the development of strength during ecdysterone supplementation and strength training [15,21]. Despite this finding of inconsistency with animal research, only a small number of scientific studies demonstrate the performance-enhancing effects of ecdysterone. For example, an increase in grip strength in rats has been reported, and a PI3K-mediated mechanism has been discussed [14]. 

In addition, our findings revealed that 1RM leg press/FFM and 1RM bench press/FFM decreased by 2.6% and 7.4%, respectively, in the PLA, whereas, in the 20E, 1RM leg press/FFM and 1RM bench press/FFM decreased by 2.5% and 1%, respectively, after the 12-week detraining period. In comparison to the PLA group (7.4% reduction), the 20E group (1% reduction) was able to maintain significantly greater upper-body muscle strength following detraining. The results of this study support a previous report whereby ecdysterone rendered a stronger anabolic effect than metandienone, even when not combined with training [36]. 

In terms of anabolic and catabolic hormones, changes in free testosterone levels (103.4% and 49.4% increases, respectively) and the fTC ratio (161.5% and 85.3% increases, respectively) were observed after the RT and detraining periods. No differences existed between the PLA and 20E groups. These findings indicate that supplementation with 20E had no direct effect on free testosterone levels or the fTC ratio during both training and detraining periods. Thus, training might be able to explain the potential effects on free testosterone levels and the fTC ratio. This result is consistent with previous studies revealing that ecdysterone has no direct effect on anabolic hormones, especially free testosterone levels post-RT [15,21]. Furthermore, there was no clear effect of 20E supplementation or training on IGF-1 levels during the RT period. Surprisingly, neither 20E supplementation nor training increased IGF-1 levels during the RT period. It has been reported that supplementation with low-dose ecdysterone (12 mg/day) increases IGF-1 levels, whereas supplementation with high-dose ecdysterone (48 mg/day) does not [15]. This finding could imply that 20E, or ecdysterone, can cause a dose-dependent increase in IGF-1 levels. For a comprehensive understanding of the effect of 20E on the expression of anabolic hormones, additional research is necessary.

Interestingly, the findings of this study indicate that 20E supplementation exerts an anticatabolic hormonal effect by significantly reducing serum cortisol levels (a 23.3% reduction) in response to RT. Although intensive RT may have a negative effect on cortisol levels, this could be countered with 20E supplementation. Cortisol is described as an inducer of catabolic processes in skeletal muscle [37], whereas IGF-1 is consistently associated with anabolic activation [38]. The decreased cortisol levels, though not the increased IGF-1 levels, as a result of supplementation with 20E explain the observation that 20E exerts an anticatabolic hormonal effect, inducing muscle hypertrophy. However, additional research will be required to confirm this assumption. 

Cortisol levels in the 20E group decreased after 12 weeks of training (23.3% reduction) and were maintained further during the 12-week detraining period (20.5% reduction). The outcomes of this study support a previous report whereby ecdysterone administration decreased corticosterone levels in male rats [19]. Nevertheless, the mechanism by which 20E reduces cortisol levels has not yet been identified. Our findings support previous findings in that 20E derived from the hard-stem byproducts of *A. officinalis* extract is a promising alternative to anticatabolic hormones. Supplementation with 30 mg/day of 20E could have an anticatabolic effect that induces muscle hypertrophy during the RT period and could prevent muscle mass loss due to detraining by reducing catabolic hormone levels during the detraining period.

Furthermore, no side effects directly attributable to 20E supplementation were demonstrable. There was no increase in biomarkers for liver or kidney toxicity. Although supplementation with 20E from *A. officinalis* hard-stem by-products did not increase muscle strength and mass during the RT period, it had a positive effect in delaying muscle mass and strength loss in young, healthy men during detraining. The potential applications of 20E could be expanded to include the prevention of muscle mass and strength loss from detraining.

### Limitations of the Study

Due to the low number of participants in this study, it was impossible to analyze the data according to mass. The statistical literature consistently cites a larger sample size as reducing variability and producing more meaningful and accurate results. The participants were instructed to maintain their normal lifestyles and refrain from regular exercise during the detraining period, yet there was no way to prevent them from engaging in additional physical activity during this period. Nonetheless, the significant decreases in a number of measurements indicate that the subjects were able to adhere to these restrictions.

## 5. Conclusions

Our findings showed that supplementing with 20E derived from *A. officinalis* hard-stem by-products did not improve muscle strength and mass more than RT alone during the RT period. Nonetheless, this intervention produced a favorable outcome among young, healthy men, as it demonstrated the ability to prevent the loss of muscle mass and strength that occurs during periods of detraining. Furthermore, 20E may have an anticatabolic effect during the RT period, as well as the ability to prevent muscle mass loss due to detraining by lowering catabolic hormone levels during the detraining period. Potential applications of 20E derived from the hard-stem by-products of *A. officinalis* extract could be expanded to include the prevention of the detraining-induced loss of muscle mass and strength in young, healthy men who experience training interruptions. 

## Figures and Tables

**Table 1:** Participants’ characteristics in the PLA and 20E groups.

	PLA (*n* = 10)	20E (*n* = 10)	*p*-Value
Age (years)	20.4 ± 1.4 (18–23)	19.8 ± 0.8 (19–21)	0.241
Height (cm)	172.6 ± 5 (164–179)	171.1 ± 4.9 (164–183)	0.509
Weight (kg)	73.5 ± 10.3 (55–96.4)	70.9 ± 9.4 (54.4–85.3)	0.571
BMI (kg/m^2^)	24.6 ± 3 (20.5–30.2)	24.2 ± 2.6 (20.2–28.8)	0.725
FM (kg)	16.9 ± 6.7 (7.9–29.9)	15.8 ± 5.8 (8–27.8)	0.698
FFM (kg)	56 ± 6.2 (43.5–67.2)	54 ± 6.5 (44.8–62)	0.485
V̇O_2_peak (mL/kg/min)	42.8 ± 9.2 (20.8–53.7)	38.4 ± 5.5 (27.4–46.8)	0.216
1RM Leg press (kg)	360 ± 61.2 (264.7–469.1)	350.6 ± 80.1 (232.3–466.8)	0.253
1RM Bench press (kg)	63.7 ± 11.1 (45–82.3)	58 ± 10.3 (41.1–78.8)	0.770

Note: Data are shown as means ± SD (range: minimum to maximum). PLA, placebo group; 20E, 20-hydroxyecdysone supplementation group; V̇O_2_peak, peak oxygen consumption; BMI, body mass index; FM, fat mass; FFM, fat-free mass; 1RM, one-repetition maximum.

**Table 2:** Body composition, body circumferences, and muscular strengths before training, after training, and after detraining in the PLA and 20E groups.

	PLA (*n* = 10)				20E (*n* = 10)				Time Effect *η*^2^ (*p*-Value)	Group × Time Interaction *η*^2^ (*p*-Value)
	Pre-Training	TR-12	DeTR-6	DeTR-12	Pre-Training	TR-12	DeTR-6	DeTR-12		
**Body composition**										
Total BM (kg)	73.7 ± 10.4	75.2 ± 10.3	74.4 ± 9.9	73.2 ± 9.4 ^b^**	71.5 ± 9.5	72.3 ± 8.9	72.5 ± 8.5	72.3 ± 7.8	0.152 (0.030) ^††^	0.081 (0.203)
Total %BF (%)	23.3 ± 6.4	22.9 ± 6.2	23.7 ± 6.6	22.6 ± 6.2	22.7 ± 5.9	20.7 ± 6.1	21.9 ± 5.5	21.7 ± 5.6	0.132 (0.053)	0.065 (0.298)
Total FM (kg)	16.9 ± 6.7	16.9 ± 6.2	17.2 ± 6.4	16.1 ± 5.6	15.8 ± 5.8	14.7 ± 5.7	15.5 ± 5.2	15.3 ± 5.1	0.076 (0.229)	0.059 (0.342)
Total FFM (kg)	56 ± 6.2	57.6 ± 6.8	56.8 ± 6.6	57.1 ± 6.2	54 ± 6.5	56 ± 5.7 ^a^*	55.4 ± 5.5	55 ± 4.9	0.278 (<0.001) ^††^	0.071 (0.257)
Arm mass (kg)	8.8 ± 1.5	9.2 ± 1.5	9.2 ± 1.8	8.9 ± 1.6 ^b^**	8.3 ± 1.4	8.5 ± 1.1	8.6 ± 1.1	8.5 ± 1.1	0.218 (0.004) ^††^	0.037 (0.556)
Arm fat (%)	21.4 ± 7.2	21 ± 7	21.2 ± 6.9	20.7 ± 6.8	19.6 ± 4.8	17 ± 5.4 ^a^*	18.6 ± 4.6	18.5 ± 4.4	0.176 (0.014) ^††^	0.105 (0.108)
Arm FM (kg)	1.8 ± 0.9	1.9 ± 0.8	1.9 ± 0.9	1.8 ± 0.8	1.6 ± 0.5	1.4 ± 0.6	1.5 ± 0.5	1.5 ± 0.5	0.073 (0.248)	0.103 (0.114)
Arm LM (kg)	6.6 ± 0.9	6.9 ± 1	6.9 ± 1.2	6.7 ± 1.1	6.4 ± 1.1	6.7 ± 0.8 ^a^*	6.7 ± 0.8	6.6 ± 0.8	0.310 (<0.001) ^††^	0.013 (0.867)
Leg mass (kg)	26.9 ± 4.1	27.2 ± 4.1	26.9 ± 3.8	26.3 ± 3.7	26.1 ± 4.1	26.1 ± 3.9	26.1 ± 3.5	25.7 ± 3.1	0.110 (0.096)	0.017 (0.819)
Leg fat (%)	22.4 ± 4.8	22.1 ± 5	23.1 ± 5.5	22.2 ± 5	22.1 ± 5.2	20.5 ± 5.4	21.4 ± 4.7	21.1 ± 4.8	0.122 (0.068)	0.064 (0.310)
Leg FM (kg)	5.9 ± 2	5.8 ± 1.9	6 ± 2	5.7 ± 1.8	5.6 ± 2	5.2 ± 2	5.4 ± 1.7	5.3 ± 1.7	0.096 (0.138)	0.037 (0.556)
Leg LM (kg)	19.8 ± 2.5	20.1 ± 2.7	19.7 ± 2.4	19.5 ± 2.5 ^b^**	19.4 ± 2.4	19.7 ± 2.3	19.5 ± 2.1	19.3 ± 1.8	0.157 (0.026) ^††^	0.026 (0.692)
**Body circumferences**										
Chest (cm)	94.7 ± 5.2	97.6 ± 5.5 ^a^**	96.4 ± 4.8	94.9 ± 4.8 ^b^***	92.2 ± 5.3	94.3 ± 4.9 ^a^*	93.8 ± 5.8	93.7 ± 4.5	0.362 (<0.001) ^††^	0.133 (0.051)
Waist (cm)	79.5 ± 7.5	80.3 ± 6.6	79.9 ± 6.4	79.5 ± 6.1	78.1 ± 5.9	78.8 ± 6.2	79.4 ± 5.2	79.3 ± 5.3	0.055 (0.376)	0.080 (0.208)
Arm (cm)	32 ± 3.6	31.6 ± 3.2	31.3 ± 3.1	30.8 ± 3.1	30.5 ± 3.3	30.7 ± 2.9	30.6 ± 2.7	30.4 ± 2.6	0.177 (0.014) ^††^	0.102 (0.119)
Forearm (cm)	28 ± 1.3	28.1 ± 1.2	28.1 ± 1.5	27.6 ± 1.3 ^b^***	27.2 ± 1.8	27.2 ± 1.5	27.8 ± 1.9	27 ± 1.4	0.236 (0.002) ^††^	0.075 (0.238)
Thigh (cm)	58 ± 5.8	60.3 ± 4.4	59.7 ± 4.8	58.7 ± 4.4 ^b^**	57.5 ± 3.8	58 ± 3.9	58.5 ± 3.7	57.2 ± 3.7	0.237 (0.002) ^††^	0.077 (0.226)
Calf (cm)	39.8 ± 3.3	40.1 ± 2.7	40.2 ± 3.3	39.6 ± 3.2	39.2 ± 3.2	39.5 ± 3	39.6 ± 2.8	39.5 ± 2.7	0.105 (0.110)	0.048 (0.448)
**Muscular strengths**										
1RM leg press (kg)	360 ± 61.2	486.9 ± 111.5 ^a^**	466.7 ± 68.4	469.2 ± 120.2	305.6 ± 80.1	448.5 ± 63 ^a^**	433.6 ± 49.7	443.3 ± 68.1	0.593 (<0.001) ^††^	0.021 (0.763)
1RM leg press/FFM	5.8 ± 1	8.4 ± 1.4 ^a^**	8.3 ± 1.2	8.2 ± 1.6	5.6 ± 1.3	8 ± 0.8 ^a^**	7.8 ± 0.7	7.8 ± 1.1	0.673 (<0.001) ^††^	0.005 (0.961)
1RM bench press (kg)	63.7 ± 11.1	74 ± 13.5 ^a^**	70.8 ± 14.3	68.3 ± 15.4 ^b^***	58 ± 10.3	68.4 ± 11.7 ^a^**	70.2 ± 13	68.6 ± 10.2	0.622 (<0.001) ^††^	0.027 (0.156)
1RM bench press/FFM	1.1 ± 0.2	1.3 ± 0.2 ^a^**	1.2 ± 0.2	1.2 ± 0.2	1.1 ± 0.1	1.2 ± 0.1 ^a^**	1.3 ± 0.2	1.2 ± 0.1	0.487 (<0.001) ^††^	0.104 (0.113)

Note: Data are shown as means ± SD. PLA, placebo group; 20E, 20-hydroxyecdysone supplementation group; TR-12, after 12 weeks of training; DeTR-6, after 6 weeks of detraining; DeTR-12, after 12 weeks of detraining; %BF, body fat percentage; BM, body mass; FM, fat mass; FFM, fat-free mass; LM, lean mass. †† indicates *η*^2^ ≥ 0.14; *, **, and *** indicate *p* < 0.05, *p* < 0.01, and *p* < 0.001, respectively. ^a^ indicates a comparison to pre-training; ^b^ indicates a comparison to after 12 weeks of training.

## Data Availability

The data used to support the findings of the current study are available from the corresponding author upon request.

## Supplementary Materials

The following supporting information can be downloaded at: https://www.mdpi.com/article/10.3390/sports11090175/s1, Figure S1: HPLC chromatogram of the standard solution of 20-hydroxyecdysone (250 μg/μL) with a retention time of 25.805 min (A) and the hard-stem by-product of *A. officinallis* at a retention time of 25.923 min (B).; Table S1: Total daily energy intake at baseline, training, and detraining for 12 weeks in the PLA and 20E groups.
