# Supplementary material for: Beneficial Effects of Asparagus officinalis Extract Supplementation on Muscle Mass and Strength following Resistance Training and Detraining in Healthy Males
Source: Sports (Basel). 2023 Sep 5;11(9):175. doi: 10.3390/sports11090175 (PMC10537221; doi:10.3390/sports11090175)
Supplement: Supplementary file 1 [file sports-11-00175-s001.zip › sports-2556190-supplementary.pdf]

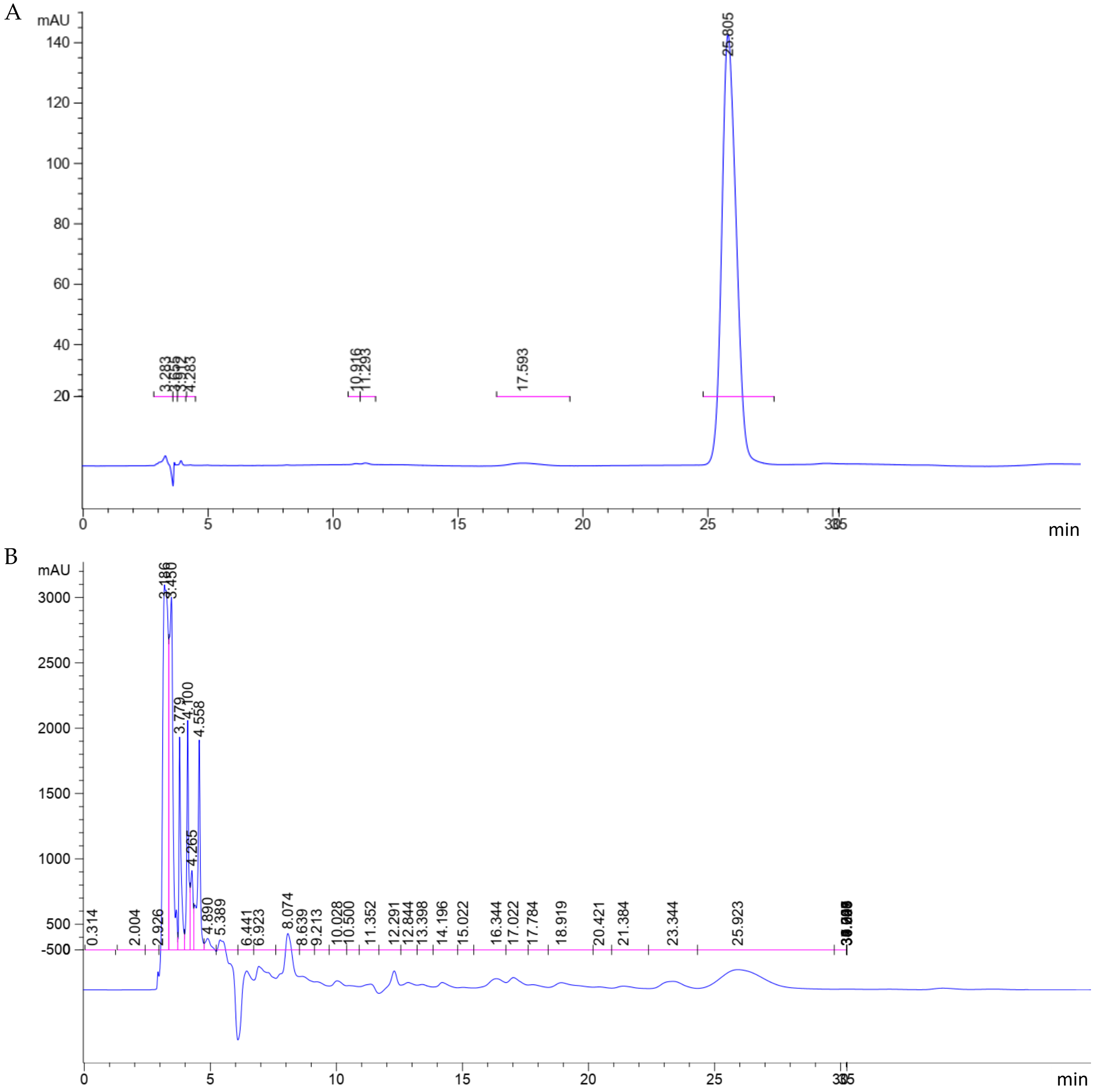

Figure S1. HPLC chromatogram of the standard solution of 20-hydroxyecdysone (250 µg/µL) with a retention time of 25.805 min (A) and the hard-stem by-product of *A. officinallis* at a retention time of 25.923 min (B).

Table S1. Total daily energy intake at baseline, training, and detraining for 12 weeks in the PLA and 20E groups.

	PLA (n = 10)			20E (n = 10)			Time effect η^2 (*p*-value)	Group× Time interaction η^2 (*p*-value)
	**baseline**	**TR-12**	**DeTR-12**	**baseline**	**TR-12**	**DeTR-12**		
Energy intake (Kcal/day)	1877.7±188.6	1961.5±138.7	1936.2±223.1	1881.0±317.9	1848.6±229.1	1829.4±219.6	0.016 (0.747)	0.082 (0.213)
Energy intake (Kcal/day/kgBW)	26.0±4.1	26.6±3.4	26.9±4.2	26.9±5.1	26.1±4.3	25.7±3.9	0.002 (0.968)	0.110 (0.124)
CHO (g/day)	235.5±22.11	248.3±14.5	243.0±30.3	235.0±39.2	233.2±29.9	231.3±27.0	0.038 (0.497)	0.080 (0.223)
CHO (g/kgBW)	3.6±0.5	3.4±0.4	3.4±0.6	3.4±0.6	3.3±0.5	3.3±0.5	0.005 (0.910)	0.099 (0.154)
CHO (Kcal)	942.05±88.5	993.3±57.8	972.0±121.0	942.1±156.6	932.8±119.7	925.0±107.8	0.038 (0.497)	0.080 (0.223)
CHO (E%)	50.2±1.9	50.7±1.4	50.17±1.6	50.1±0.9	50.5±1.2	50.6±0.7	0.022 (0.676)	0.017 (0.739)
PRO (g/day)	88.8±17.1	97.5±9.6	97.8±22.6	89.6±17.5	87.5±12.5	86.7±12.3	0.035 (0.527)	0.105 (0.137)
PRO (g/kgBW)	1.2±0.3	1.3±0.2	1.3±0.3	1.3±0.3	1.2±0.3	1.2±0.2	0.011 (0.824)	0.100 (0.149)
PRO (Kcal)	355.8±68.5	384.0±38.4	380.2±76.8	358.4±70.0	350.2±50.0	346.8±49.2	0.024 (0.642)	0.100 (0.174)
PRO (E%)	18.8±2.1	19.9±0.7	19.5±2.1	19.0±1.0	19.0±2.0	18.9±1.2	0.034 (0.538)	0.036 (0.512)
FAT (g/day)	64.4±6.1	64.3±6.0	64.9±4.4	64.5±10.7	62.9±9.0	61.9±8.1	0.024 (0.644)	0.042 (0.465)
FAT (g/kgBW)	0.9±0.1	0.9±0.1	1.0±0.1	0.9±0.2	0.9±0.2	0.9±0.1	0.065 (0.299)	0.109 (0.126)
FAT (Kcal)	579.8±55.0	578.3±54.1	584.0±39.5	580.4±96.2	565.63±81.1	557.5±73.2	0.024 (0.644)	0.042 (0.465)
FAT (E%)	30.9±1.5	29.5±1.2	30.3±1.7	30.9±1.0	30.6±1.5	30.5±1.4	0.178 (0.029)	0.091 (0.181)

Note: Data are shown as means ± SD. PLA, placebo group; 20E, 20-hydroxyecdysone supplementation group; TR-12, after 12 weeks of training; DeTR-6, after 6 weeks of detraining; DeTR-12, after 12 weeks of detraining; CHO, carbohydrate; PRO, protein; FAT, fat.
